# Enhanced Gas-Sensing Performance of GO/TiO_2_ Composite by Photocatalysis

**DOI:** 10.3390/s18103334

**Published:** 2018-10-05

**Authors:** Eunji Lee, Doohee Lee, Jaesik Yoon, Yilin Yin, You Na Lee, Sunil Uprety, Young Soo Yoon, Dong-Joo Kim

**Affiliations:** 1 Materials Research and Education Center, Auburn University, Auburn, AL 36849, USA; ezl0020@auburn.edu (E.L.); dzl0051@auburn.edu (D.L.); jzy0063@auburn.edu (J.Y.); 2 Department of Mechanical Engineering, Auburn University, Auburn, AL 36849, USA; yzy0037@auburn.edu; 3 Department of Chemical and Biological Engineering, Gachon University, Seongnam 13120, Korea; ynl87@gc.gachon.ac.kr (Y.N.L.), benedicto@gachon.ac.kr (Y.S.Y.); 4 Department of Physics, Auburn University, Auburn, AL 36849, USA; szu0007@auburn.edu

**Keywords:** wearable gas sensor, graphene oxide, titanium dioxide, graphene-metal oxide composite, photocatalysis, photoreduction

## Abstract

Few studies have investigated the gas-sensing properties of graphene oxide/titanium dioxide (GO/TiO_2_) composite combined with photocatalytic effect. Room temperature gas-sensing properties of the GO/TiO_2_ composite were investigated towards various reducing gases. The composite sensor showed an enhanced gas response and a faster recovery time than a pure GO sensor due to the synergistic effect of the hybridization, such as creation of a hetero-junction at the interface and modulation of charge carrier density. However, the issue of long-term stability at room temperature still remains unsolved even after construction of a composite structure. To address this issue, the surface and hetero-junction of the GO/TiO_2_ composite were engineered via a UV process. A photocatalytic effect of TiO_2_ induced the reduction of the GO phase in the composite solution. The comparison of gas-sensing properties before and after the UV process clearly showed the transition from n-type to p-type gas-sensing behavior toward reducing gases. This transition revealed that the dominant sensing material is GO, and TiO_2_ enhanced the gas reaction by providing more reactive sites. With a UV-treated composite sensor, the function of identifying target gas was maintained over a one-month period, showing strong resistance to humidity.

## 1. Introduction

A gas sensor is a device that identifies the presence and amount of target gases. The detection of gas molecules is extremely important for many purposes—such as environmental monitoring, air and water quality control, agricultural condition monitoring, and food safety— to prevent disastrous accidents from gas emission, normally as part of a safety system [1,2,3]. Recently, as the demands of flexible and wearable electronics increase, wearable sensors have emerged as a new interest for smart sensing applications [4,5]. By combining ubiquitous computing and smart textile technology, a high performance sensor with wearability has been developed to be adapted in various applications such as the health care system [6,7]. To advance wearable gas sensors, various chemiresistive materials have been explored. Conventionally, metal oxides such as SnO_2_, ZnO, and TiO_2_ have been utilized in chemiresistive sensors due to their numerous merits such as low cost, stability, short response time, and long lifetime [8,9]. Since metal oxide sensors operate at high working temperatures, such sensors are improper for wearable electronics [10]. In accordance with wearable applications, 2D nanostructured materials have been explored as alternative materials instead of metal oxide. Due to their distinct physical and chemical characteristics, 2D nanostructured materials have successfully decreased the sensing temperatures up to room temperature (RT, 25 °C) and their sensing performance has been progressed [11,12]. Among many 2D nanomaterials, there has been much research toward graphene and graphene oxide (GO) for gas-sensing applications owing to their large specific surface area and potential binding sites [13,14]. Modified GO has been more frequently utilized than pure GO. Since oxygen functional groups of GO play the role of both reaction sites and reduction electrical conductivity, the number normally has been balanced through reduction processes [15,16,17]. GO or reduced GO (rGO) has demonstrated RT-sensing ability with good sensing performance. However, the GO sensors operating at RT show the major drawbacks of the sluggish or irreversible recovery and lack of long-term stability, which should be further refined [16].

One approach to improve the performance of a GO sensor was to incorporate GO with metal oxides [18,19]. Similar to metal oxide composites, hybridizing 2D materials and metal oxide can be postulated to have syntagmatic benefits for sensing performance. By compositing GO and metal oxide nanoparticles, synergistic effects combined from each material might be facilitated, resulting in enhanced gas-sensing performance as well as reduced cost [20]. Recent literature has studied various metal oxide nanoparticles as a second material in the graphene matrix, and the representative metal oxides are SnO_2_, ZnO, and WO_3_ [21,22]. Depending on the choice of the second material, diverse sensing performances, such as gas response and selectivity, were improved. However, a clear explanation of the sensing behavior of the composite has not been established [21]. Therefore, further study should be implemented to understand the sensing mechanism and physics behind performance improvement. Although the photocatalysis of GO/TiO_2_ has been well investigated [23,24], relatively less effort has been dedicated to the investigation of gas-sensing properties of GO and TiO_2_ hybridization. Few papers have reported on RT gas-sensing results of rGO decorated TiO_2_ through a hydrothermal method [25,26]. Furthermore, few studies have investigated the gas-sensing properties of GO/TiO_2_ composite combined with photocatalytic effect. Hence, it would be worthwhile to explore the gas-sensing properties of the GO/TiO_2_ composite in company with the photocatalytic effect of TiO_2_. 

In this presented study, the GO/TiO_2_ composite gas sensors were fabricated by a simple solution method, and gas-sensing properties of the composite were explored with various reducing gases at RT. For further investigation associated with photocatalysis, UV was exposed to the GO/TiO_2_ composite solution, and the gas-sensing properties of the UV-treated GO/TiO_2_ composite were examined. Based on the sensing results, a possible sensing mechanism to explain the composite’s properties, along with photocatalysis, is discussed in detail.

## 2. Materials and Methods

### 2.1. GO/TiO_2_ Nano-Composite Synthesis

GO powder (0.5–2.0 μm, ACS Materials Company, Pasadena, CA, USA) with 50 mg was dispersed in 15 mL ethanol and stirred for 30 min. The GO solution was mixed with 2.5 mM of TiO_2_ nanoparticles (size < 21 nm, a mass fraction of 80% anatase/a mass fraction 20% of rutile, Sigma-Aldrich, St. Louis, MO, USA) and stirred until the mixed nanopowder was completely dispersed in ethanol. After vigorous sonication, the solution was irradiated by UV (ELC-500 UV curing chamber, 30 W/${\mathrm{cm}}^{2}$) for 2 h.

### 2.2. Gas-Sensing Device Fabrication

Gas-sensing devices were constructed on pliable polyimide films. To eliminate surface pollutants of the film, it was cleaned with ethanol and distilled water, then dried with an air gun. On the cleaned sensor platform, a stainless-steel shadow mask with an array of interdigitated electrodes was taped to the pattern sensor circuit. Platinum was direct current sputtered on the masked polyimide film with a thickness of about 100 nm. Then, 10 mg of the prepared solution was dropped on the body of interdigitated electrodes of a polyimide substrate and dried overnight in a desiccator. 

### 2.3. Material Characterizations and Gas-Sensing

The JEOL JSM-7000F scanning electron microscope (SEM) was utilized to observe the morphology of the synthesized GO nanosheets and TiO_2_ nanoparticles. Ultraviolet-visible (UV-vis) spectroscopy was employed to characterize the optical property of the composite (Ultra spec 2100 pro, Harvard Bioscience, Inc., Holliston, MA, USA) in the wavelength range of 400–800 nm. Structure and surface condition of the composite were investigated using X-ray diffraction (XRD) and room temperature micro-Raman spectroscopy. XRD measurements were conducted with a scan speed of 2°/minute on the dried GO nanosheets and TiO_2_ nanoparticle films. The homemade Raman system is equipped with a Jobin Yvon spectrometer and a thermal electrically-cooled charge coupled device (CCD) detector (2048 pixels by 512 pixels). The Raman spectroscopy was carried out in back scattering geometry using 441.563 nm line (80 mW) from a He-Cd laser. To examine gas-sensing properties, the sensor electrode coated with the composite was connected to the resistance measurement equipment (Keithley 2400 source meter) in the test chamber. The synthetic air was consistently flown into the chamber with the amount of 40 sccm of nitrogen and 20 sccm of oxygen gas. At the same time, the target gas was delivered with the aid of 40 sccm of nitrogen as passing through the liquid analyte. The resistance of the sample was recorded in the presence or absence of the target gas under the continuous synthetic air atmosphere by using a customized LabVIEW program. The gas response was calculated as the ratio of $\left|R_{a}-R_{g}\right|/R_{a}$, where $R_{g}$ is the resistance of the film under the target gas, and $R_{a}$ is the resistance of the film under the ambient air.

## 3. Results and Discussion

### 3.1. Characterization Results

**Figure 1:**
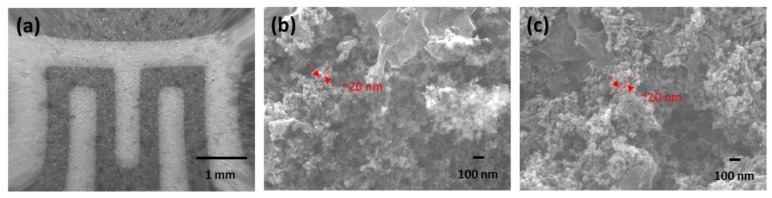
SEM images of GO/TiO_2_ composite in (**a**) low magnified view and high magnified view (**b**) before UV irradiation and (**c**) after 2 h UV irradiation.

The morphology of the hybridized GO nanoflakes and TiO_2_ nanoparticles was observed by SEM. Figure 1a shows the dried film of the GO/TiO_2_ composite after drop casting onto the sensor platform of the polyimide film. The two mixed materials uniformly filled up the gap of the interdigitated electrode. In a magnified view of Figure 1b, it is observed that GO nanoflakes and TiO_2_ nanoparticles were tangled. The size of TiO_2_ nanoparticles was confirmed to be around 20 nm. The wrinkled morphology of GO sheets was due to sp^3^ bonding of the oxygen-containing groups on the GO surface. Figure 1c shows the magnified view of GO/TiO_2_ composite film after 2 h UV treatment in solution. The randomly mixed GO nanoflakes and TiO_2_ particles from UV-treated composite showed similar morphology with the sample before UV irradiation. When GO is coupled with TiO_2_, the carboxyl group on the GO surface could bond with the hydroxyl group on the TiO_2_ surface, and the charge transfer under UV irradiation could result in photoreduction of GO [27]. The combination of GO and TiO_2_ was sustained after UV irradiation, which suggests that the coupled structure is unaffected by UV energy [28]. Besides, the size of TiO_2_ nanoparticles had not grown or congregated, which is usually observed by heat processes [29]. 

**Figure 2:**
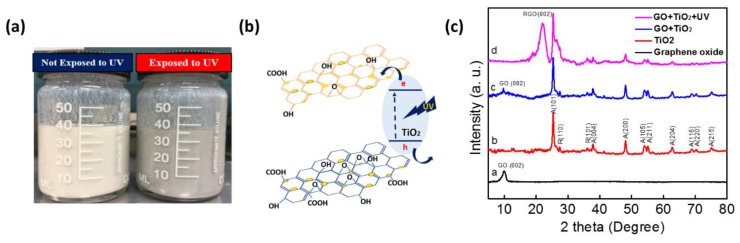
(**a**) The photograph of GO/TiO_2_ composite before and after UV exposure. (**b**) The schematic diagram of GO/TiO_2_ composite under the UV effect. (**c**) XRD spectra of the prepared (a) GO, (b) TiO_2_, GO/TiO_2_ composite (c) before, and (d) after UV exposure.

Figure 2a is the photograph of the composite solution where GO nanosheets were mixed with TiO_2_ nanoparticles in ethanol. The left bottle is the mixed solution before UV irradiation, and the right bottle is that after 2 h UV irradiation. Since both TiO_2_ and GO carry a surface charge in suspension, they can easily disperse in the solution and be stable for several hours. After several days the mixture sank, especially for the UV-treated solution. However, it can be suspended again by mild ultra-sonication or stirring. After UV exposure, the obvious change in color from white to gray was observed, which indicates that GO was photo-catalytically reduced (rGO). This color difference originates from the restoration of sp^2^ bonds from sp^3^ bonds by removing oxygen functional groups on the surface of GO nanosheets [30]. Figure 2b presents the photocatalytic reduction mechanism of GO with TiO_2_ nanoparticles under UV irradiation. When UV light, which has a photon energy larger than the band gap of TiO_2_, was exposed to the composition solution, electrons and holes in TiO_2_ nanoparticles were separated. In the presence of ethanol, the holes are transferred to ethanol, which generates ethoxy radicals of ethanol. Corresponding electrons are crossed to GO, eliminating certain amounts of oxygen functional groups [31]. The following Equations (1) and (2) indicate the photocatalytic reduction process [28].

(1) $${\mathrm{TiO}}_{2}+\mathrm{hv}\;\to {\mathrm{TiO}}_{2}\left(h+e\right){\;\mathrm{in}\;C}_{2}H_{5}\mathrm{OH}\to {\;\mathrm{TiO}}_{2}\left(e\right)+{\;C}_{2}H_{4}{\mathrm{OH}}^{\cdot }+{\;H}^{+}$$

(2) $${\mathrm{TiO}}_{2}\left(e\right)+\mathrm{GO}\;\to {\;\mathrm{TiO}}_{2}+\mathrm{rGO}$$

The successful photo-reduction of GO with the presence of photocatalyst was confirmed by X-ray diffraction (XRD) in Figure 2c. A distinct peak of GO (2Θ = 10°) [32] and anatase/rutile TiO_2_ (JDPDS 21-1272, 21-1276) are clearly observed in Figure 2c. In the XRD pattern of TiO_2_, the average size of nanoparticles calculated from the Scherrer equation (FWHM = 0.136°) was about 23 nm, which is consistent to the SEM result. For the simple mixture of GO and TiO_2_, each peak from GO and TiO_2_ were collectively shown, however, the typical peak of GO seems a small hump due to the crystallized peak of TiO_2_. After UV exposure to GO and TiO_2_ solution, a new diffraction peak belonging to rGO is observed at 2Θ = 24°, and a peak of GO centered at 2Θ = 10° disappeared [33]. This result indicates that the UV treatment can be an effective method for the reduction of GO to rGO.

**Figure 3:**
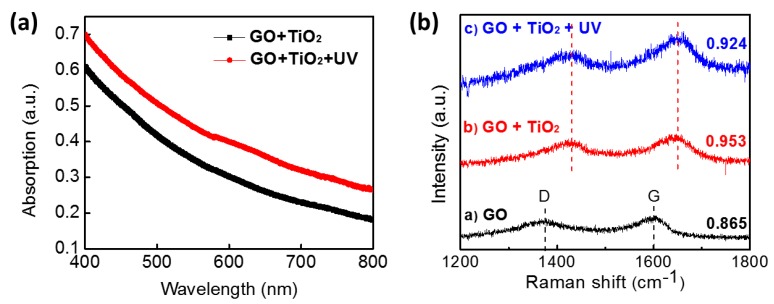
(**a**) UV-vis absorption spectra of GO/TiO_2_ composite before and after UV irradiation. (**b**) Raman spectra of (a) GO, (b) GO/TiO_2_ composite, and (c) GO/TiO_2_ composite with UV irradiation.

UV-visible (UV-vis) spectroscopy and Raman were employed to further investigate the effect of UV by characterizing of the composite. Figure 3 depicts the UV-vis spectra of the composite with the absence and presence of UV irradiation in the range of 400 to 800 nm. For both samples, UV absorption was decreased as the wavelength increased. With UV-treated solution, absorption spectra were generally increased in the whole range. This indicates that the electronic conjugation was restored in the graphene sheet, which corresponds to the color change of the sample where UV-treated composite looked darker in Figure 2a. Figure 3b shows the Raman spectra of (a) GO, (b) GO/TiO_2_ composite, and (c) GO/TiO_2_ composite after UV irradiation. Raman spectroscopy can reveal information regarding the local structure of the composite and the quality of carbon structure. The main feature in the Raman spectra of graphene composite was assigned in the D band at 1375 cm^−1^ and G band at 1598 cm^−1^ [34]. Normally, the D band corresponds to graphite structure as attributing to in-plain vibration of sp^2^ bonded carbon domain [35]. The G band is attributed to sp^3^ hybridized carbon bonds mostly due to the presence of structural defects as suggesting disordered crystalline carbon structure [36]. In this regard, the intensity ratio of the D band to the G band can describe an extension of the disorder or defects in the carbon domain. In Figure 3b, the value of $I_{D}/I_{G}$ ratio is 0.865 in GO, 0.953 in the composite, and 0.924 in UV-treated composite. In GO film, oxygen functional groups along with defects impute to sp^3^ hybridization on sp^2^ graphene matrix. Compared to the ratio of pure GO, the $I_{D}/I_{G}$ ratio of the composite increased, which indicates that the composite has more sp^3^ hybridized bonds. The increase might be associated with the introduction of TiO_2_ nanoparticles. Possibly, when incorporating GO with TiO_2_, some TiO_2_ nanoparticles might create Ti-O-C bonds in GO plain, resulting in increased $I_{D}/I_{G}$ ratio [36]. After UV irradiation, $I_{D}/I_{G}$ ratio was decreased more compared to that without UV since the sp^2^ bonds of GO were restored by photocatalysis of TiO_2_. Along with the UV-vis result, this confirmed that GO was photo-reduced by TiO_2_ catalysis and the surface condition of the composite was modified. Meanwhile, the peak shift of both D and G bands were obviously observed after hybridizing GO and TiO_2_ as shown in Figure 3b. It is indicated that a peak shift can be observed when materials are under stress and that a blue shift (higher wavenumber) can be induced by compression [23,37]. As a result, blue shift of the bands can be interpreted that carbon atoms in GO are under compression due to structural change induced by TiO_2_ nanoparticles [38]. Correspondingly, the length of chemical bonds in GO can be modified by adding TiO_2_ into GO. However, D and G band position were similarly maintained after UV treatment, which depicts no further structure change and chemical bonds.

### 3.2. Gas-Sensing Performance

**Figure 4:**
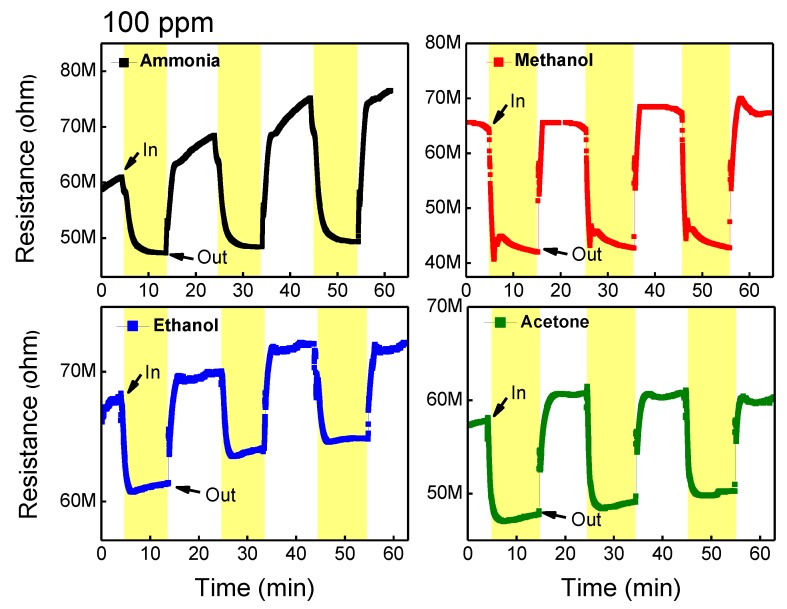
Gas-sensing results of GO/TiO_2_ composite film without UV irradiation toward 100 ppm ammonia, methanol, ethanol, and acetone gas bubbling at room temperature (25 °C).

The gas-sensing properties of GO/TiO_2_ composite were investigated in Figure 4. This film was simply fabricated by a powder mix in the solvent without any further treatment. Various reducing gases such as ammonia, methanol, ethanol, and acetone gases were introduced with the concentration of 100 ppm, and the gas-sensing behavior of the composite was explored at RT. For the gas-sensing test, the composite film was periodically exposed to target gases for 10 min followed by a flow of ambient air where 40 sccm of nitrogen and 20 sccm oxygen were mixed. As shown in Figure 4, the initial resistance of the composite was very high, ranging from 6[WL1] 0~70 MΩ because of the insulating property of TiO_2_ at RT. The hybridized film exhibited n-type sensing behavior to the reducing gases. Upon introducing the target gas, the resistance of the film was dropped and then recovered to the initial resistance without the gas as showing n-type sensing behavior. Considering the RT-sensing ability of 2D nanomaterials and the insulating property of metal oxide, it is expected that GO would play a role of dominant sensing material and networking TiO_2_ nanoparticles [25]. On the other hand, TiO_2_ might play a catalytic role to attract gas molecules by lowering the activation energy [25,39]. Detecting methanol, ethanol, and acetone with high response can be attributed to the catalytic effect of TiO_2_, while only ammonia was identified by pure GO film.

**Figure 5:**
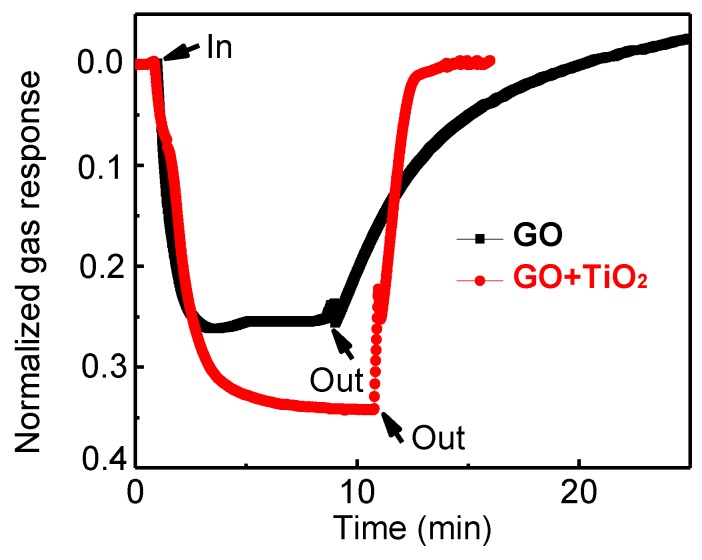
Comparative gas-sensing results of pure GO and GO/TiO_2_ composite toward 100 ppm ammonia gas bubbling at room temperature (25 °C).

To evaluate the gas-sensing performance of the GO/TiO_2_ composite, gas-sensing results were compared to pure GO film in Figure 5. The gas response was calculated as the ratio of $\left|R_{a}-R_{g}\right|/R_{a}$, where $R_{g}$ is the resistance of the film under the target gas, and $R_{a}$ is the resistance of the film under the ambient air. The gas response of pure GO film was found to be 0.23, and that of the GO/TiO_2_ composite was 0.38. By combining 2D nanomaterial with metal oxide nanoparticles, the gas response against ammonia was improved. The recovery time of the composite was also compared to that of pure GO against ammonia gas. It was defined as the time to recover 90% of resistance before gas injection. As shown in Figure 5, the recovery time of GO towards ammonia is around 15 min, whereas that of the composite is 2 min. Even though the base resistance drift of both films existed, recovery time was obviously reduced in the composite structure. The enhanced response and reduced recovery time of the composite can be attributed to the creation of n-n junction at the interface and the catalytic sensitization of TiO_2_ nanoparticles [40].

**Figure 6:**
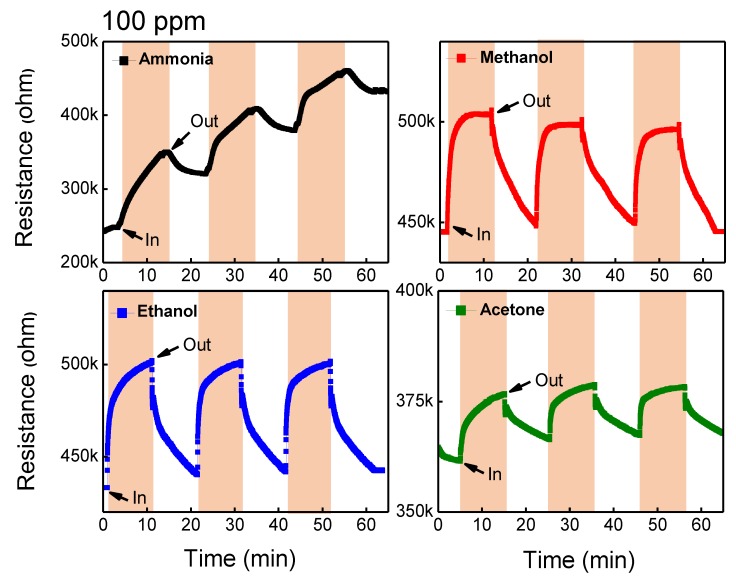
Gas-sensing results of UV irradiated GO/TiO_2_ composite toward 100 ppm ammonia, methanol, ethanol, and acetone gas bubbling at room temperature (25 °C).

During the fabrication process, UV light was irradiated to the composite solution for 2 h, then the gas-sensing properties of the dried TiO_2_/GO composite film were investigated. The same reducing gases with the concentration of 100 ppm were utilized, and the sensing properties of the UV-treated composite were explored at RT in Figure 6. Since GO in the composite was photocatalytically reduced by UV irradiation, the initial resistance of the composite dropped to a 300–400 kΩ range. In contrast to the composite sample, the UV-treated sample exhibited p-type gas-sensing behavior to the same reducing gases due to GO reduction. In the reverse way of n-type-sensing behavior, the resistance was increased when target gas was injected and then decreased when it disappeared. In the middle of the two conditions, the 1 h UV-treated composite sensor did not react to any target gases, and this originates from the cancellation of the opposite sensing behavior during n-p transition. Such sensing behavior transition provides empirical evidence for an ambipolar characteristic of GO or rGO [41,42]. By controlling the oxygen functional groups, GO can have either a p-type or n-type sensing behavior [42,43]. Furthermore, the sensing behavior conversion concludes that the dominant sensing material of the composite at RT would be GO or rGO, and TiO_2_ acted as reactive sites to improve sensing performance.

**Figure 7:**
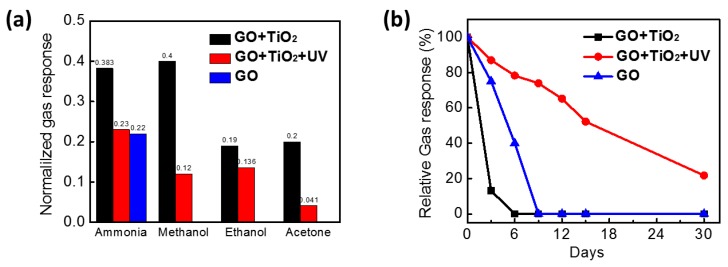
(**a**) The compiled gas response and (**b**) the long-term response of UV-treated and non-treated GO/TiO_2_ composite film to ammonia gas.

The gas-sensing performance of the hybridized samples was evaluated by incorporating the photocatalysis effect. The gas response of the GO/TiO_2_ composite to various gases was calculated and plotted in Figure 7a. The gas responses of the composite to ammonia, methanol, ethanol, and acetone were 0.383, 0.4, 0.18, and 0.2, respectively. These values of gas responses toward volatile organic compounds (VOCs) were higher than pure GO, rGO, and novel metal decorated rGO due to the synergistic effect of hybridization [14,44,45]. In the case of UV-treated composite, the general gas responses to reducing gases decreased to 0.2, 0.12, 0.136, and 0.041. It has been well studied that the oxygen functional groups, such as hydroxyl and epoxy groups of GO, are essential to possess high gas responses to target gases because they can easily absorb the gas molecules [12]. Since the number of oxygen functional groups on GO nanosheets were reduced from photo-reduction, the chance of interaction between functional groups and target gases was decreased resulting in a lower gas response. Although the overall gas response of the photo-reduced composite was reduced compared to pure composite, this result is quite comparable to pure GO film. Figure 7b presents the long-term stability of both composite gas sensors over a month. The GO/TiO_2_ composite showed a higher gas response to ammonia than the UV-treated composite, however, it maintained the function for a short time. The initial resistance of the composite sample was 70 MΩ at first and increased to over 100 MΩ after 3 days, resulting in no response to gases. The increased resistance is due to the fast oxidation of the composite as showing susceptibility to humidity. On the other hand, the UV-treated composite demonstrated that the sensor was able to work over a month at RT. Even though the response to ammonia gradually declined for a month, the response was reliable. This improvement might be due to the controlled amount of oxygen functional groups which easily reacted with water molecules in ambient atmosphere.

### 3.3. Gas-Sensing Mechanism

**Figure 8:**
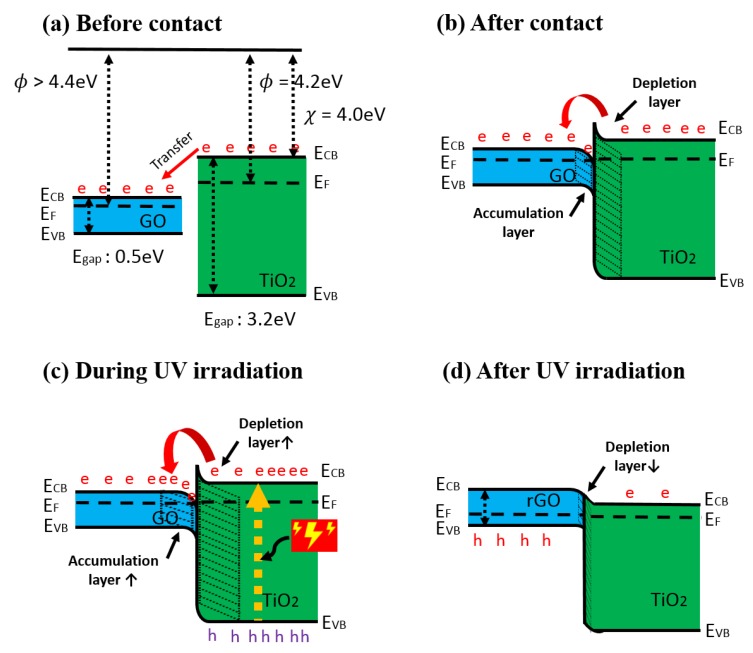
The proposed band diagram of GO/TiO_2_ composite (**a**) before contact, (**b**) after contact, (**c**) during UV irradiation, and (**d**) after UV irradiation. (e: electron, h: hole).

The improved gas-sensing performance of the composite could be attributed to the synergistic effect of GO and TiO_2_ nanoparticles. Figure 8a,b illustrates the proposed band diagram of the GO/TiO_2_ composite before and after contact. It is reported that the work function of graphene is 4.4 eV [23] and oxidation increases the work function of carbon-based materials such as CNT and fullerene, as well as graphene [46]. In this work, the work function of GO was speculated to be higher than 4.4 eV, and it was estimated to be around 4.7 eV [47]. Contact between GO nanosheets and TiO_2_ nanoparticles formed an n-n junction at the interface, and charge carriers were transferred from TiO_2_ to GO. This resulted in an accumulation layer and a depletion layer as illustrated in Figure 8b [48]. Due to the Schottky barrier, the depletion layer of TiO_2_ was thicker, and the number of electrons in GO was increased at the interface. At RT, the main contribution of the resistance change in the composite sample was caused by the reaction between oxygen functional groups on GO and gas molecules. However, with excessive electrons transferred to GO, the electrostatic force, such as Van der Waals force, may enhance physisorption of the polar gases, which leads to enhanced gas response and faster recovery time compared to pure GO film. In addition, negatively charged oxygen adsorbates ($O_{2}^{-})$ on the surface of TiO_2_ would support gas reaction by lowering activation energy to attract gas molecules as catalytically active sites during the sensitization process [26]. Structurally, TiO_2_ nanoparticles helped to prevent GO from agglomeration and increase the number of reaction sites, which provide a higher chance to react with target gas [49,50]. As a result, the GO/TiO_2_ sensor was able to measure various reducing gases, as well as ammonia gas, and improve gas performance, such as higher response and faster recovery time.

Nevertheless, the long-term stability of the composite was degraded due to susceptibility to humidity in the ambient atmosphere and high initial resistance. The large number of oxygen functional groups of GO and the oxygen adsorbates ($O_{2}^{-})$ of TiO_2_ film cause dynamic interaction with humidity or contaminants, resulting in increased resistance. It has been studied that the ambient oxygen and moisture are easily absorbed on the surface of GO by oxygen functional groups, such as hydroxyl, and increase resistance [51]. Because the reactive sites become occupied by moisture under the ambient atmosphere, the function of the composite sensor cannot be maintained for a long time.

In order to improve stability of the composite sensor, the composite solution was UV treated, and its surface and junction was engineered during the UV irradiation. As mentioned in Equations (1) and (2), electron-hole pairs were photo-generated in TiO_2_, and excited electrons were transferred to GO under the exposure of UV light. During the UV irradiation, the accumulation layer of the GO and depletion layer of the TiO_2_ could be temporarily increased at the interfaces as described in Figure 8c. The excessive electrons shifted to GO were employed to restore π bonds of carbon structure by eliminating oxygenated groups and to remove moisture on the surface of GO. Accordingly, GO became photo-reduced, and the p-n junction might be formed with decreased junction width at the interface as shown in Figure 8d. By integrating TiO_2_ with GO, photolysis of TiO_2_ was encouraged by injecting electrons into GO, and the recombination of electron-hole pairs in TiO_2_ was effectively prevented [33].

As a result, the gas detection ability of the UV-treated composite sensor was preserved over a month, and sensing behavior was altered from n-type to p-type. The number of oxygen functional groups of GO in the composite was adjusted by the photocatalysis of TiO_2_. Thus, the hydrophilic property of GO was curtailed, and relative hydrophobic property of rGO suppressed moisture absorption [52]. Moreover, the amount of oxygen adsorbates ($O_{2}^{-})$ on TiO_2_ was diminished by the photo-active hole in Equation (1), resulting in the reduction of moisture attraction [53]. The photo-induced effect on gas performance remained for over a month at the cost of a slight sacrifice of gas response.

## 4. Conclusions

The room temperature gas-sensing performance of the GO/TiO_2_ composite prepared on flexible polymeric film was enhanced by the effect of photocatalysis of TiO_2_ under UV. By incorporating TiO_2_ to GO, the surface condition of GO was tailored, and Schottky barriers were created at the interface. As a result, detection of various reducing gases was enabled with the aid of TiO_2_ nanoparticles, and the recovery time was quite reduced compared to pure GO film. However, the long-term stability still remains as an unsolved problem due to susceptibility to humidity. To solve the stability issue of the sensor, the GO/TiO_2_ composite solution was UV treated, and the surface and hetero-junction of the composite was modulated. Due to the photocatalytic effect of TiO_2_ under UV irradiation, electrons were transferred to GO, resulting in the photo-reduction of GO. Thereby, the majority charge carrier of the reduced GO was switched to holes, and p-n junction was produced at the interface instead of Schottky contact. The performance of the hybridized composite sensor was comparatively evaluated with the same reducing gases. Understandably, the gas-sensing behavior of the hybridized sensor was converted from n-type to p-type, which confirms that the dominant sensing material of the composite is GO, and TiO_2_ acts as a catalyst. Although a slight reduction of gas response in the UV-treated sensor was observed due to the changed amount of the oxygen functional groups in GO via photolysis of TiO_2_, the function to identify target gas was maintained over a one-month period, showing strong resistance to humidity. This result suggests great potential of a GO/TiO_2_ composite sensor with the photocatalytic effect for realizing multiple gas detection at RT as well as long-term stability.
